# Grand challenges and living labs: toward achieving the Sustainable Development Goals

**DOI:** 10.3389/fpubh.2023.1242138

**Published:** 2023-07-24

**Authors:** Andreea Molnar, Robert Lepenies, Ann Borda, Sonja Pedell

**Affiliations:** ^1^Department of Computing Technology, Swinburne University of Technology, Melbourne, VIC, Australia; ^2^Karlshochschule International University, Karlsruhe, Germany; ^3^Melbourne School of Population and Global Health, University of Melbourne, Parkville, VIC, Australia; ^4^Department of Information Studies, University College London, London, United Kingdom; ^5^Swinburne Living Lab, Swinburne University of Technology, Melbourne, VIC, Australia

**Keywords:** grand challenges, living labs, Sustainable Development Goals, global opportunities, scale, transformation

## 1. Introduction

In 2015, the United Nations (UN) established a set of Sustainable Development Goals (SDGs) and an Agenda for sustainable development to achieve these goals by 2030 (1). The Agenda^1^ was adopted by all member states of the UN. The SDGs are a collection of 17 interlinked global goals^2^ designed to be a “blueprint to achieve a better and more sustainable future for all”. Through its targets, the SDGs encourage the international community and all stakeholders to promote sustainable actions for people, planet and prosperity—tackling issues from multidimensional poverty, education, and the promotion of health and wellbeing, while addressing climate change, biodiversity and protecting the environment. The SDGs also encourage partnerships to maximize the value created by collaborations and the interconnectedness of the goals. The SDGs have become a global framework that is guiding action for public and private actors alike: whether national, regional or local sustainability plans in policy; sustainability reporting for corporations or campaigns for NGOs —increasingly, sustainability is framed in terms of the SDGs. In this article, we explore the role of living labs in achieving SDGs—their potential and limitations. Achieving the SDGs means also achieving a reorientation of how we think about sustainability: leaving no one behind, thinking intersectionally, and simultaneously considering the synergies and trade-offs when creating transformations toward a more just and sustainable world.

## 2. SDGs and living labs—A problem of scale?

The role of living labs as a contributor to the SDGs is particularly relevant through its social impact process of partnerships and innovative solution development. In a systematic review of living lab literature (2), the identification of living labs and sustainable development was noted as a growing intersection of activity, focusing on the support of holistic solutions and general support of sustainability through a continuum of learning and development that considers socio-economic, educational and environmental impacts.

Living labs are spaces (either physical or virtual) through which stakeholders meet to collaborate on finding solutions to a complex issue (2). They could be used to generate ideas, develop and/or test solutions (3). Living labs are not only used by researchers but they could be also activities started by citizens, non-profit organizations or industry (4).

The SDGs have a global ambition and are the result of decades of deliberation at the international level about sustainability involving an unprecedented number of stakeholders. And while much of Agenda 2030 is directed to policy makers representing nation states and calls for top-down policy change, the SDGs explicitly call for the involvement of everyone to achieve the “transformation of our world”. At the same time, sustainability must be rooted in local, concrete actions and bottom-up activities–which often take a more experimental form. It is in this tension between global ambition and local necessity that living labs can play a vital bridging role.

Considering the complexity of SDGs' goals, and the multitude of stakeholders needed to be involved in order to address a single goal, living labs seem to be a highly pertinent tool through which stakeholders can come together, generate ideas and work collaboratively. Living labs can also support testing solutions before being deployed on a large scale.

The role of living labs as a contributor to the SDGs is particularly relevant through its social impact process of partnerships and innovative solution development. In a systematic review of living lab literature (2), the identification of living labs and sustainable development was noted as a growing intersection of activity, focusing on the support of holistic solutions and general support of sustainability through a continuum of learning and development that takes into account socio-economic, educational and environmental impacts.

The Open Living Lab Days 2018 hosted by the European Network of Living Labs (5) reinforced the potential of living labs to contribute to the SDGs in domains such as health, energy and education, among others. Sustainability and sustainable development are integrated into the activities of individual labs, such as urban living labs with a focus on the green economy, environmental health, and achieving net zero efforts, for instance (6, 7).

Larger networks similarly share a number of sustainability goals. The Australian Living Labs Innovation Network (8) incorporates organizational values encompassing a circular economy, water waste and energy, natural environment and climate change resilience. The multi-national iSCAPE living labs network have centered on advancing air pollution remediation strategies and solutions, and the UnaLab cities consortium is working toward developing sustainable urban communities through the implementation of nature-based solutions. The Living Laboratories Initiative in Canada is an example of a sectoral network focusing on new approaches to agricultural innovation addressing agri-environmental issues (9).

Massachusetts Institute of Technology Office of Sustainability has made exemplary strides in transforming the campus into a living lab responding to the challenges of a changing planet (10). However, it is noted in selected studies that such sustainability efforts by universities can be implicit (11), and the concept of sustainability itself can be oversimplified (12). Notwithstanding, there is evidence that universities are beginning to acknowledge that the greatest SDG challenges can only be solved by systems thinking and finding solutions for a shift toward interdisciplinary collaboration to achieve this (13, 14). Achieving the SDGs, therefore, implies a change in mindsets, behaviors and policy.

## 3. Sustainable Development Goals and Grand Challenges

The concept of “Grand Challenges” has been adopted by policymakers and research organizations to frame and communicate their respective agendas (15). These challenges also represent more than ordinary research questions or priorities, they are outcomes at global scale which capture the public imagination (16). In general, Grand Challenges initiatives are characteristically anchored by a set of foundational principles (17) like the characteristics of the living lab approach. Engagement through multi-stakeholder involvement, experimentation through openness, implementation of solutions in the real world and transformation—the focus on having impact. These fundamental pillars are evidenced in the Grand Challenges in Global Health initiatives launched by the Bill & Melinda Gates Foundation in 2003 which continue to address solutions to health problems in the developing world across 15 identified challenges (18). Governments have engaged in grand challenges, such as Grand Challenges Canada supported by the Canadian government and based upon the Gates Foundation model to develop solutions to critical health and development challenges in disadvantaged communities. It is also a vehicle to empower innovators “who are closest to the world's health challenges because they have the knowledge and are best positioned to develop lasting solutions”.

Sectoral areas have defined their futures through key challenges like the Grand Challenges for Social Work initiative led by the American Academy of Social Work and Social Welfare (17). Universities have similarly adopted the model as part of their strategic vision. University College London (UCL) has established a six-grand challenge agenda to develop cross-disciplinary collaborations related to solving some of the world's most pressing problems (19). These are linked to achieving the SDGs across global health, human wellbeing, cultural understanding, sustainable cities, justice and equality, and transformative technology.

The UCL Grand Challenges reflect the six Transformation areas developed to organize SDG interventions in which each transformation is intended to engage different levels of government, industry and civil society, to facilitate targeted problem-solving (20). This new way of thinking about tackling “wicked” challenges has its roots in what can be termed “mission-oriented research and innovation” which can potentially provide a more effective and crucial link between the Grand Challenges of the SDGs and the multidisciplinary research and innovation knowledge needed to tackle them (21).

Horizon Europe has recently opted to use missions for its research and innovation program for 2021–2027 (22). These missions support Commission priorities, such as the European Green Deal, Beating Cancer, Climate Adaptation Strategy, and Europe's Rural Areas. A key stakeholder cross-cutting approach is the incorporation of citizen engagement in events, online discussions, and social media polls, for instance, at the Conference on the Future of Europe.^3^

## 4. Limitations and opportunities

In reflecting on the positioning of living labs in a Grand Challenge context, there is much potential for living labs to demonstrate their intrinsic value in accelerating SDG progress. The SDGs in their breadth and scope require the collective intelligence that living labs have fostered—that is an understanding in practice that the resources of intelligence can be brought together and shared, from localized insights and inventions from people on the ground, to data and evidence (23). Not least, Grand Challenges are nearly impossible to accomplish without coordinated, collaborative, and co-created innovation (24). As Gilbertson et al. (13) acknowledge, stakeholder support is vital to the success of partnerships addressing complex problems.

### 4.1. About direct contributions to the SDGs

Living labs are recognized as progressive platforms for fostering innovation and strengthening collaborative partnerships from bottom-up (25–27). They are often networked which means solutions can be distributed and scaled more quickly from local to national to global levels (26). These networked ecosystems more readily support an innovation lifecycle of piloting, implementation and evaluation (27). However, studies on the sustainability directions of living labs have been oversimplified in comparative studies (12). When considering the situated nature of sustainability research (28), living labs are relevant study points and drivers of real-world initiatives enabling the investigation of sustainability in place (12). For instance, living lab approaches have been considered in the design and implementation of Nature-Based Solutions as a means of reducing the exposure to natural disasters, such as increased flooding in changing climates (29).

In contributing formally to the SDGs, there are two main ways in which living labs can be involved: first, by implementing or supporting measures that lead to improvement of SDG implementations (27), second, by contributing to SDG reporting and monitoring. Now, living labs mostly contribute in the first way: their actions might lead to solutions that contribute to the achievement of the underlying aims or goals of the SDGs (e.g., by providing a healthier urban climate in a specific context, or by fostering equitable partnerships in a given location), but do so in broad—and difficult to measure—terms. This can also be done indirectly, e.g., by holding policy makers or business corporations to account as part of networks in which SDG topics are discussed and/or political movements organized.

The second opportunity might be for living labs to officially contribute to the SDG reporting and monitoring mechanisms. All countries (in their voluntary national reviews at the UN level), but also many cities and communes report their progress on SDG achievement by using SDG indicators. There are limited examples in which citizen science initiatives or living labs have formally partnered up with mandated statistical authorities to aid in these efforts (30). These have been closely aligned with directives on air quality monitoring, for example (30, 31). Given the technical nature of sustainability reporting, it is understandable that living labs often do not have the expertise to directly contribute to specific monitoring mechanisms. There are, notwithstanding, opportunities for living labs to be involved in place-based data collection and/or as part of data hubs which collectively contribute, such as through the UN Habitat urban observatories or OECD program on city region-based approaches to tackling SDGs (32).

### 4.2. Achievability

Critically, the timeline for achieving the SDGs is 2030. There is an urgent acknowledgment on government and political agendas of the need to further advance collective work on the SDGs (12). The Social Progress Index (33) indicates that the COVID-19 pandemic may have delayed achievement of the SDGs by several decades and may have even reversed some efforts. According to the UN SDG 2019 report (34), progress toward SDGs had already been lacking in several areas. The COVID-19 pandemic has magnified these largely unmet areas, such as racial and cultural inequities in access to healthcare and education, and a widening gap of gender-based inequities globally (35).

In 2019, the UN Development Programme (UNDP) supported the establishment of a global network of accelerator labs to tackle some of the most pressing and underachieved SDGs in the global south. The network covers 115 countries and nearly 100 labs addressing goals such as Goal 5: Gender equality, Goal 13: Climate action, Goal 15: Life on land, Goal 17: Partnerships for the goals (36).

The AI4Good Foundation (ai4good.org) is an example of an emerging technology organization supporting AI applications to help accelerate the achievement of SDGs with the use of shared datasets, such as Global Forest Watch under Goal 15: Life on Land, and Ocean Tracking Network under Goal 14: Life Below Water.

It is difficult to ascertain the extent to which living lab initiatives formally align with specific SDG indicators or are represented in the official monitoring system (UN SDG Indicators). At present, national governments have the primary responsibility for monitoring the SDG indicators in which each SDG indicator has one or more custodians (e.g., a UN agency) who are responsible for identifying the data sources that can contribute to each SDG indicator. This may entail practical limitations for living labs, due to potential resourcing requirements, for instance. A recent study (37) has explored the fact that information is still lacking regarding the current and potential contributions of citizen science collected data to the SDG indicator framework. The same study noted that both indirect and direct contributions are valuable assets toward achieving targeted SDGs, but the contribution process can be highly context-dependent in different countries.

Looking into the future, living labs have the potential to contribute to the SDGs. Using the SDGs as a framework allows living labs to map out the synergies and trade-offs between different dimensions of sustainability. This will avoid siloed thinking and will embrace the holistic and systemic attitudes that are at the core of the SDGs. It is this type of thinking that could break down—at the very local level, and in concrete contexts—the global ambitions for sustainability into concrete, contextually rooted actions in communities.

## 5. Final reflections

With the global push for achieving the SDGs in less than a decade, there remains a wide opening for living labs to significantly contribute as individual and collaborative networks both in formal and informal ways. The process of tackling SDGs highlights their complex nature.

Optimizing the process has elicited targeted and mission-oriented agendas alongside the broader Grand Challenges approach. What is shared is the transformative and global opportunity for living labs to leverage their relationships, technology, and communities, to collectively enable the most positive and sustainable impacts to the benefit of humanity and the world we share.

## Author contributions

All authors listed have made a substantial, direct, and intellectual contribution to the work and approved it for publication.

## Footnotes

^1^ https://sdgs.un.org/2030agenda

^2^ https://sdgs.un.org/goals

^3^ https://futureu.europa.eu/
